# Prevalence and associated factors of neurodevelopmental disability among infants in eastern Uganda: a population based study

**DOI:** 10.1186/s12887-019-1769-z

**Published:** 2019-10-24

**Authors:** Gertrude Namazzi, Helena Hildenwall, Paul Mubiri, Claudia Hanson, Christine Nalwadda, Margaret Nampijja, Angelina Kakooza-Mwesige, Peter Waiswa, James K. Tumwine

**Affiliations:** 1 Makerere University School of Public Health, College of Health Sciences, Mulago Hill Road, P. O. Box, 7072 Kampala, Uganda; 2 Global Health-Health Systems & Policy, Public Health Sciences, Karolinska Institutet, Stockholm, Sweden; 3 Astrid Lindgren Children’s Hospital, Karolinska University Hospital, Stockholm, Sweden; 4 Department of Disease Control, London School of Hygiene and Tropical Medicine, London, England; 5 MRC/Uganda Virus Research Institute & LSHTM Research Unit, Entebbe, Uganda; 6 Department of Paediatrics and Child Health, School of Medicine, College of Health Sciences, Makerere University, Kampala, Uganda

**Keywords:** Neurodevelopmental disability, Prevalence and associated factors, Infants, Eastern Uganda

## Abstract

**Background:**

Neurodevelopmental disability (NDD) is increasingly acknowledged as one of the important causes of disease burden in low income countries. None the less, there is a dearth of data on the burden of NDD and its determinants in these settings. We aimed to establish the prevalence and factors associated with NDD among infants in Eastern Uganda.

**Methods:**

We assessed 487 infants aged 9–12 months within Iganga-Mayuge Health Demographic Surveillance Site in Eastern Uganda using the Malawi Developmental Assessment Tool. The tool has four domains: gross motor, fine motor, language and social domains. An infant failed a domain if she/he failed more than two parameters of the expected at his/her age.

We interviewed mothers on factors that could influence the infants’ neurodevelopmental outcomes. Data were analysed using STATA version 14. We used odds ratios and 95% confidence intervals to assess statistical significance of associations.

**Results:**

Of the 487 infants, 62(12.7%) had an NDD in at least one of the domains. The most affected was social behaviour where 52(10.7%) infants had an NDD. Severe impairment was seen among 9(1.8%) infants with NDD in either three or four domains.

Factors associated with NDD at multivariate logistic regression included: parity of more than three children (aOR = 1.8, 95% CI: 1.02–3.18); failure to cry at birth (aOR = 3.6, 95% CI: 1.46–9.17) and post-neonatal complications (aOR = 4.15, 95% CI: 1.22–14.10). Low birth weight, immediate and exclusive breast feeding were not significantly associated with NDD.

**Conclusion:**

We found a high NDD burden among infants particularly in the social behaviour domain. To optimise the socio-neural development of infants, programs are needed to educate and work with families on how to engage and stimulate infants. Existing immunisation clinics and community health worker strategies provide an excellent opportunity for stemming this burden.

## Introduction

Globally, for the past decade there have been significant strides to curb neonatal and infant mortality, albeit with an increased risk of neurodevelopmental disabilities (NDD) [1–3]. More than 80% of these disabilities are in low and middle income countries (LMIC) [4, 5] where 90% of the world’s children live [6]. None the less, there is paucity of accurate data characterising developmental disability in these settings. The NDD include deficiencies in motor functions, socio-emotional behaviour, sensory function, attention, memory and language during the early period of a child’s growth [7]. These may result in profound limitations in learning and realising full developmental potential with subsequent constraints in social and economic development of communities and nations at large [4, 5].

According to the bio-ecological systems theory of early child development, biological and environmental risk factors have a complex interaction that may result in NDD [8]. The environmental risk factors include: limited social interactions, poverty, and one’s surroundings [9]. Socio-emotional development of children is shaped by family relationships and parental practices [10]. Children learn by observing those around them and through social referencing. Loving and positive relationships coupled with active stimulation and encouragement result in an environment that builds confidence of the child in his/her ability to communicate and relate with others. However, these relationships may be compromised by the number of children in the family [11, 12].

Biological risk factors on the other hand, comprise of insults to the unborn baby, including poor health status and nutritional deficiencies during the prenatal period, child birth, infancy and early childhood [9].

There is consistent evidence showing that newborn babies suffering from conditions such as sepsis/infections, asphyxia (defined as failure to cry at birth at community level), premature birth, low birth weight and pathological jaundice have a higher likelihood of NDD [2, 13–15]. This risk may be compounded by the three delays in seeking care: delay in making a decision due to ignorance and cultural practices; delay in reaching the health facilities and delay in receiving quality care at health facilities due to supply side challenges [16].

In Uganda, more than a quarter of births occur at home or with the aid of unskilled traditional birth attendants who are not considered effective to reduce the burden of ill-health but pose a higher risk of intrapartum complications and resultant NDD [17, 18]. The situation is worse in rural areas with delays in identification, management and follow up care of vulnerable infants in the neonatal and the post-neonatal period. In addition, mothers may not seek care for their sick infants from health facilities due to limited physical access to care or because of cultural beliefs and use of traditional remedies. While the use of Community Health Workers (CHWs), locally known as Village Health Teams (VHTs), is wide spread in the country, utilization of child health services is still limited with potential negative consequences on child development [19].

The Uganda national policy framework on early child development gives guidance for access to quality services for all children from conception to eight years. These services include education, nutrition, child protection, health care, family and community support and empowerment. Despite the existence of this policy framework, implementation of some essential interventions is still inadequate [20]. For instance programmes that engage, and empower families and communities to foster early child stimulation and development are limited. This might be due to lack of national statistics on the burden, pattern and risk factors of early child NDD.

To inform policies and programming for appropriate responses and actions in the country and areas with similar settings, this study aimed at assessing the prevalence of NDD and associated factors among infants aged nine to twelve months of age in a health demographic site in Eastern Uganda. This study focused on health care factors that can potentially influence neurodevelopmental outcomes during pregnancy, child birth, neonatal and the post-neonatal period. In addition, we also assessed the demographic and socio-economic factors of the parents.

## Methods

### Study area and design

We conducted a cross-sectional study in December 2017 in the Iganga/Mayuge Health Demographic Surveillance Site (HDSS) in Eastern Uganda. The HDSS has 65 villages with a population of about 86,000 people who are mostly (80%) rural and practice subsistence farming. Most of the people in the study area are *Basoga,* the third largest ethnic tribe in Uganda. Generally, in this context, there is preference for large families, and child rearing is mostly the responsibility of the mother. However, due to the many responsibilities of women, children are usually left to play with fellow children, and parents are rarely involved in active stimulation of their children. There is also an extended family system with controlling parenting practices by multiple family members, like the rest of the country [21]. The total fertility rate is 5.4 with average annual births of around 2000 babies of whom 11% are low birth weight or preterm babies [22]. The neonatal and infant mortality rates in 2015 were 30 and 60/1000 live births respectively compared to national average rates of 27 and 43/1000 live births [17]. The site is served by one general hospital which offers comprehensive Emergency Obstetric and Newborn Care, and 12 lower level health facilities, which offer basic maternal and newborn care services. There are, also, community health workers, who have been trained in basic maternal and newborn care and support the tracking of pregnancies and their outcomes. The site has been active since 2005 when a population baseline census was conducted and all households were given identification numbers. The site conducts bi-annual censuses using trained field assistants during which surveillance data is updated. The surveillance data includes: births, deaths, migration, and pregnancy outcomes [22].

### Study participants and procedures

We used data of the surveillance round done between March and May 2017. Five hundred seventeen infants born between January and March 2017 (who were expected to be nine to twelve months in December 2017) were identified. Information captured from the surveillance round data included: the villages, household heads, and mothers’ names, names of the babies and dates of birth. Household identification numbers were also recorded and used to help in physical identification of the homesteads during data collection.

We carried out neurodevelopmental assessments using the Malawi Developmental Assessment Tool (MDAT) in December 2017. The MDAT tool has four developmental domains: Gross motor, Fine motor, Language and Social behaviour. The tool was created and validated to assess neurodevelopmental outcomes of children in rural African setting from birth to six years. The validation tests of the MDAT tool indicated high sensitivity and specificity to identify NDDs of 97 and 82% respectively [23]. Similarly, the overall reliability was very good with a *kappa* of more than 0.75.

In our study, the assessment involved getting a parental report, observation with a checklist, and direct administration to the child. Table 1 shows examples of the parameters that were assessed under each MDAT domain. For each parameter the child scored 1 if it was able to perform and a 0 if not. The child was assessed on several parameters in each domain until s/he failed six parameters consecutively then the assessment of that domain was stopped. An infant failed a domain if s/he failed more than two parameters of the expected at his/her age. The assessments were done by seven nurses who received a five day training in use of the MDAT tool.

**Table 1:** Examples of parameters of Neuro-developmental assessment using the Malawi Developmental Assessment Tool (MDAT)

Developmental domain	Method of assessment	Examples of items/parameters assessed
Gross motor	Parental report	Crawls, Pulls self to stand
	Observation	Sits without support Able to stand if holding onto things
	Direct administration	Walks using both hands of someone
Fine motor	Parental report	Transfers objects from one hand to another
	Observation/direct administration	Can use a neat pincer grasp to pick up object between thumb and fore finger Puts blocks or stones in and out of a plastic tea cup in imitation
Language skills	Parental report	Understands when being told ‘no’ or being cautioned Shakes head or does something to indicate No
	Observation report	Makes double syllable sounds, tata, dada, mama Says two other words other than mama, dada, e.g. cup, cow, spoon
	Direct administration	Follows one stage command such as ‘give me the cup’
Social behaviour	Parental report	Helps by putting hands out to be washed Indicates in some way the need for poo or pee
	Observation	Frolics with mother/caregiver in response to being played with
	Direct administration	Stretches to be picked Drinks from a cup by self

In addition to assessment using the MDAT tool, the infants were evaluated on other neurodevelopmental parameters such as sight, hearing and whether the infant had had any convulsions. We placed a dangling object in front of an infant to assess whether he/she could track and reach out. Similarly, observations were made on whether the child could turn towards the direction of a rattling noise. In case of a history of convulsions, we ruled out any associated fevers to enable differentiation of febrile convulsions from convulsions due to neural impairment. We also measured the head circumference of infants using a tape measure, and took their body weights using Seca scales.

The mothers were interviewed on socioeconomic and demographic characteristics, and health care practices which could potentially affect the neurodevelopment of the infant such as antenatal care (ANC) attendance, vaccination with tetanus toxoid, uptake of sulfadoxine pyrimethamine to prevent malaria, and ferrous sulphate and folic acid tablets. We also included delivery care practices (place of delivery, mode of delivery, whether there was a skilled attendant at birth or not, the gestational age (GA) at birth of the child, and birth outcome), and postnatal practices (cord care, keeping the baby warm, immediate and exclusive breastfeeding, as well as health seeking care practices for the infant). In addition, we asked for a history of post-neonatal complications defined as any danger sign (inability to feed well, convulsions, lethargy/unconsciousness, chest in-drawing and grunting, hypothermia, and high body temperature) after the neonatal period.

### Data management and analysis

Data from the MDAT and on risk factors as assessed in the interviews with the mothers were processed (double entered with quality checks) using EPIDATA version 3.2 and exported to STATA version 14 for analysis.

Descriptive statistics were generated on socio-demographic characteristics, care practices of the mothers during pregnancy, delivery and postnatal period, and on weight and head circumference of the infants. We generated Z-scores for weight for age and head circumference for age using Zanthro techniques [24]. Z-scores for weight for age were used to assess the nutrition status of infants. The prevalence of NDD was generated by obtaining the percentages of infants who failed at least one of the MDAT domains. Multivariable logistic regression was conducted to identify factors associated with NDD. Variables yielding a *p*-value = 0.2 in the univariate analysis, together with factors known a priori to be predictors of NDD (Father’s occupation as proxy for socio-economic status, maternal age, maternal education, breast feeding practices, and tetanus toxoid to the mother) were included in multivariable analysis. The model was refined by backward elimination using the change in log likelihood ratio of successive models with a significance level of 0.05. Variables were tested for collinearity by examining correlation coefficients, and if found to be related one was dropped.

## Results

We assessed 487 babies out of the targeted 517 in Iganga/Mayuge HDSS. Thirty infants could not be accessed: 7 were out of the age range, 3 had died and 20 had either shifted, or were not at home on three repeated visits. Out of the 487 babies assessed 144 (29.6%) were aged nine months, 116 (23.8%) were 10 months, 126 (25.9%) were 11 months, and 101 (20.7) were 12 months. The mean weight of infants at time of data collection was 8.5 kg (range: 4.5-12.5 kg; SD = 1.1 kg). The weight for age Z-scores had a normal distribution, with 5.0% below − 2.0 Z score. Similarly, the head circumference had a normal distribution of the Z scores, with a mean of 45.4 cm, SD = 1.4 cm (range: 39.0–50.3 cm). There were no significant sex differences among the infants: 250 (51.3%) were male.

We found 62 (12.7%) infants with neurodevelopmental disability in at least one of the domains. The most affected domain was the social behaviour where 52 (10.7%) infants had a NDD followed by the language domain with 20 (4.1%) affected children. Severe impairment, defined as NDD in at least three or all the four domains, was seen in 9 (1.85%) infants. Eleven (2.3%) babies, had NDD in two domains. Gross motor and Fine motor were equally affected at 1.8% (9/487). Five babies (1.03%) were reported to have had non-febrile convulsions. None of the babies had visual or auditory disabilities.

At bivariate analysis, socio-demographic factors independently associated with NDD included: parity of the mother of more than three children (OR = 1.87, 95% CI: 1.09–3.21) (Table 2). Mother’s age, level of education, and employment status of the father were not significantly associated with NDD. Similarly, antenatal care practices like ANC attendance, uptake of sulfadoxine pyrimethamine and ferrous sulphate/folic acid tablets to prevent malaria and anaemia respectively, were not associated with NDD (Table 3). Reported complications during pregnancy (High blood pressure, anaemia, fevers, vaginal bleeding) were also not associated with NDD. Delivering outside hospital (at home or primary level health facility) (OR = 1.84, CI: 1.06–3.18), infant failing to cry at birth (OR = 3.27, CI: 1.42–7.5), and experiencing post-neonatal complications (OR = 3.5, CI: 1.08–11.79) were associated with NDD (Table 3). Furthermore, if the mother reported that the infant had been ill more than three times since birth the odds of NDD increased to more than twice (OR = 2.36, CI = 1.28–4.38).

**Table 2:** Distribution of neurodevelopmental disability (NDD) by socio-demographic characteristics among infants in Iganga-Mayuge HDSS

Characteristic	Infants without NDD *N* = 425 *n* (%)	Infants with NDD *N* = 62 *n* (%)	COR (95% CI)	*p* value
Socio-demographic Characteristics				
Age of mother (years)				
< 20	52 (12.2)	6 (9.7)		
20–29	264 (62.1)	38 (61.3)	1.25 (0.50–3.10)	0.763
30+	109 (25.6)	18 (29.)	1.43 (0.54–3.82)	
Marital status				
Single/separated	52 (12.2)	3 (4.8)	2.74 (0.83–9.06)	0.129
Married	373 (87.8)	59 (95.2)		
Level of education of mother				
None, Primary, or O-level secondary (< 11 years of school)	392 (92.2)	60 (96.8)	0.39 (0.09–1.69)	0.291
Advanced level (> 11 years of School)	33 (7.8)	2 (3.2)		
Employment of father				
Salaried	72 (16.9)	8 (12.9)		
Business	130 (30.6)	29 (46.8)	2.01 (0.87–4.62)	0.040
Others	223 (52.5)	25 (40.3)	1.01 (0.43–2.33)	
Mother’s parity				
1–3	284 (66.8)	32 (51.6)		
4+	141 (33.2)	30 (48.4)	**1.88 (1.10–3.23)**	0.019

*COR* Crude odds ratio

**Table 3:** Distribution of neurodevelopmental disability by antenatal, delivery, and neonatal characteristics, among infants in Iganga-Mayuge HDSS

Characteristics	Infants without NDD	Infants with NDD	COR (95% CI)	*p*-value
ANC Attendance				
1–3 times	162 (38.1)	17 (27.4)	1.63 (0.90–2.94)	0.103
4+	263 (61.9)	45 (72.6)		
IPT/Fansidar*				
Yes	386 (90.8)	58 (93.5)	0.68 (0.24–1.98)	0.634
No	39 (9.2)	4 (6.5)		
Tetanus toxoid				
Yes	404 (95.1)	59 (95.2)	0.99 (0.28–3.38)	0.970
No	21 (4.9)	3 (4.8)		
Complications during pregnancy				
No	175 (41.2)	24 (38.7)		
Yes	250 (58.8)	38 (61.3)	1.11 (0.64–1.91)	0.710
Perinatal Factors				
Place of delivery				
Hospital	222 (52.2)	23 (37.1)		
Health Centre, private clinic,etc. (Not hospital)	203 (47.8)	39 (62.9)	1.85 (1.07–3.21)	0.026
Mode of delivery				
NVD	363 (85.4)	57 (91.9)		
Breech & C/S	62 (14.6)	5 (8.1)	0.51 (0.19–1.33)	0.234
Duration of labour				
< 24 h	323 (76.0)	49 (79.0)	0.84 (0.43–1.61)	0.599
> 24 h	102 (24.0)	13 (21.0)		
Perinatal complications				
None	389 (91.5)	59 (95.2)	0.55 (0.16–1.84)	0.454
Yes	36 (8.5)	3 (4.8)		
Neonatal and post-neonatal characteristics				
Sex of Baby				
Male	218 (51.3)	32 (51.6)	0.99 (0.58–1.68)	0.963
Female	207 (48.7)	30 (48.4)		
Birth weight				
> 2.5 kg	350 (95.6)	46 (90.2)	1	
< 2.5 kg	16 (4.4)	5 (9.8)	2.38 (0.83–6.79)	0.190
Unknown	59 (13.9)	11 (17.7)	1.42 (0.69–2.89)	
Cried at birth				
Yes	374 (94.7)	49 (79.0)		
No	21 (5.3)	9 (15.5)	3.27 (1.42–7.50)	0.025
Unknown	30 (7.1)	4 (6.5)		
Home visit after delivery				
Yes	136 (32.0)	20 (32.3)	1.01 (0.57–1.79)	0.968
No	289 (68.0)	42 (67.7)		
Immediate breast feeding at birth				
No	100 (23.5)	12 (19.4)	1.28 (0.66–2.50)	0.466
Yes	325 (76.5)	50 (80.6)		
Exclusive Breast feeding				
Yes	105 (24.8)	12 (19.4)		
No	320 (75.2)	50 (80.7)	1.37 (0.70–2.67)	0.352
Neonatal danger signs				
None	211 (49.6)	28 (45.2)		
Yes	214 (50.3)	34 (54.8)	1.19 (0.70–2.04)	0.509
Post-neonatal complications				
None	64 (15.1)	3 (4.8)		
Yes	322 (75.7)	54 (87.1)	3.58 (1.10–11.80)	0.036
Don’t know	39 (9.2)	5 (8.1)	2.73 (0.60–12.10)	
Number of times the baby fell sick since birth				
< 4	182 (42.8)	15 (24.2)		
4+	231 (54.4)	45 (72.6)	2.36 (1.28–4.38)	0.006
Unknown	12 (2.8)	2 (3.2)	2.02 (0.41–9.88)	
Admission to facility				
No	313 (73.6)	44 (71.0)		
Admitted	112 (26.4)	18 (29.0)	1.14 (0.63–2.06)	0.656

Fansidar* = sulphadoxine/pyrimethamine; ***COR*** **Crude odds ratio;** ***NDD*** **Neurodevelopmental disability**

Using multivariable logistic regression, we found three factors associated with NDD. These included: parity of mother of more than three children (aOR = 1.80, CI: 1.02–3.18), infant failing to cry at birth (aOR = 3.60, CI: 1.46–9.17) and post-neonatal complications (aOR = 4.15, 95% CI: 1.22–14.10) (Table 4).

**Table 4:** Multivariate logistic regression of key variables associated with Neuro-developmental disability among infants in Iganga/Mayuge HDSS

Covariate		COR (95% CI)	AOR (95% CI)
Mode of delivery	Normal	1	
	Caesarean/breech	0.51 (0.19–1.33)	0.20 (0.04–1.09)*
Maternal education	O Level and below	1	1
	A Level & above	0.39 (0.09–1.69)	0.58 (0.12–2.73)
Father’s occupation	Salaried	1	1
	Business	2.01 (0.87–4.62)	2.03 (0.82–5.07)
	Others	1.01 (0.43–2.33)	0.90 (0.36–2.24)
Mother’s parity	1–3	1	1
	4+	1.88 (1.10–3.23)**	1.81 (1.03–3.19)**
Cried at birth	Yes	1	1
	No	3.27 (1.42–7.54)**	3.66 (1.46–9.17)***
	Don’t know	1.02 (0.34–3.01)	4.02 (0.59–27.47)
Exclusive breast feeding for six months	Yes	1	1
	No (Gave other feeds)	1.37 (0.70–2.67)	0.62 (0.30–1.27)
Post-neonatal complications	No	1	1
	Yes	3.58 (1.10–11.80)**	4.15 (1.22–14.11)**
	Don’t know	2.73 (0.60–12.10)	2.42 (0.52–11.31)
Weight at birth	< 2.5 kg	2.37 (0.83–6.79)	2.54 (0.79–8.12)
	2.5+ kg	1	1
	Unknown	1.42 (0.69–2.89)	1.27 (0.59–2.72)

*N* = 484; LR chi2 [12]=34.84, *P*-value< 0.0005

*p < 0.1, **P < 0.05, ***P < 0.001, *COR* Crude odds ratio, *AOR* Adjusted odds ratio

The prevalence of Low birth weight (< 2.5 kg) was 5% (21/417), and NDD distribution among them was not different from those who had normal weight (*p* = 0.190) (Table 3). Most (87%) of the infants had been weighed and their birth weights were either reported by their mothers or noted from the discharge card. Of the 433 mothers who gave the LNMP, only 4(0.9%) infants had been delivered at GA less than 37 weeks. Immediate and exclusive breast feeding for at least six months were found to be almost universal, and were not significantly associated with NDD. In addition, almost all, 463(95.1%) mothers of the infants had received tetanus toxoid vaccination during pregnancy. Similarly, 96.5% of the infants had up-to-date immunization.

## Discussion

We report a high prevalence of neurodevelopmental disabilities in the studied setting, based on the MDAT tool, in the domain of social behaviour. The key associated factors were: post-neonatal complications, failure of the infant to cry at birth or birth asphyxia, and parity of more than three children. The prevalence of 12.7% NDD among infants in this study is higher than the global prevalence of 8.4% reported in the 2016 Global Burden of Disease data [25]. Our study prevalence is also higher than that reported in a study done in Kenya [11] of 2.9%, and one in Ethiopia [26] of 2.7%. However, these studies involved children less than five years of age and older children whereas we focus on infants. It is known that children with severe NDD are more likely to die early in life. Other reasons for the difference could be due to the use of different assessment tools such as the Ten Question Questionnaire, while in our study we used the MDAT tool, which also involves a physical assessment of the children, hence is more likely to capture even mild to moderate cases.

Developmental disability prevalence figures for LMIC have been reported to range from as low as 0.4 to 45.2% [26–28]. The wide variation could be due to differences in health care systems, different definitions of NDD, diverse ages of children assessed, and various tools used for measurement. Thus, there is a need for standardised assessment tools and definitions of NDD. In addition, different studies focus on different domains for assessment. In the current study, we found fewer cases of severe NDD, which could be as a result of the high perinatal mortality rates in Uganda, where the very sick neonates and early preterms (who are more likely to develop severe NDD) do not survive [29].

The results of this study are different from those reported in a study conducted in 16 LICs where impairments in language were more common than in any other domains [28]. More studies have shown a variability in the functions impaired. For example in a study conducted among Ethiopian infants the commonest (71%) disability was in the sensory (hearing) domain [26], while in the Kenyan studies it was mostly the cognitive impairment [11, 30]. It appears therefore that these differences are a result of variations in the way NDD are measured.

With regard to NDD associated factors, our findings show that both the biological and environmental factors (as highlighted under the bio-ecological systems theory) were significantly associated with NDD. While post-neonatal complications and failure of the infant to cry at birth may be considered biological factors, there could also be an environmental component related with challenges in the health systems readiness to offer quality services due to various delays in care seeking. On the other hand, the mother’s parity of more than three children is more of an environmental factor, related with social and economic demands on the family, which limit adequate child care and nurturing.

Infants with post-neonatal complications had a higher likelihood of developing neural disability (*p* = 0.036). This is in line with what was reported among Ethiopian infants in whom a third of the disabilities occurred after the neonatal period in infancy. Similar to a Nepalese study, [31], complications during the neonatal period were, unexpectedly, not associated with disability possibly due to high neonatal mortality of vulnerable newborns in Uganda [17]. Failure to cry at birth which points to intrapartum complications highlights challenges in quality of delivery/birth care services. Maulik and Darmstadt [27] argue that most developmental disabilities are preventable if quality health services are given promptly during the prenatal, natal, and postnatal period. Although there has been an improvement in institutional deliveries in Uganda from 38% in 2006 to 73% in 2016, maternal and neonatal outcomes have not changed much which might be an indication of limitations in quality of service provision [17, 32]. In a bid to address the poor neonatal outcomes, the Ministry of Health in Uganda, through its strategic plan is scaling up basic neonatal care services at Primary Health Care centres and comprehensive neonatal care at higher level health centres and district hospitals nationwide. In addition, there are plans for provision of intensive neonatal care at regional level [33]. As a result, more newborns will be saved; however, if the quality of services is not put into consideration, the rate of NDD among survivors is likely to increase [34]. Therefore, this calls for vigilance in strengthening quality of care services while improving access, not only during the intrapartum and neonatal period but also throughout infancy.

Unexpectedly, LBW was not found to be associated with NDD in our study. However, we had limited power to indicate an association due to limited number of LBW babies in our sample which could be due to survival bias. Similar results were found in a study of the prevalence of cerebral palsy in Uganda by Kakooza-Mwesige et al., [29]; and by other researchers in countries with high neonatal mortality [11, 31]. On the other hand, a systematic review by Mwaniki et al., 2013, found neonatal insults (prematurity, neonatal infections, and others) as significant risk factors for long-term NDDs [35]. These findings call for further prospective longitudinal studies.

In the current study we found an association between mothers’ parity and presence of NDD which could be due to challenges in social-interactive caregiving practices. Similar findings were reported by a study from Kenya [11] where parents having more than five children was significantly associated with neurological impairment. Parents/caregivers with many children may be less likely to give the desired attention, support and stimulation of their children hence the high disability in social behaviour domain in the current study. Socio-emotional development is important in control of one’s feelings, building relationships with others, and ability to explore and learn about the surroundings [10]. This kind of development which starts right after birth is nurtured through daily interactions with parents and other people around the children [10, 36]. Thus, parents and families need more support on how to effectively interact with their infants/children, in the course of daily care, to foster socio-emotional development [37]. In Uganda, the extended family system, often polygamous, with several children in the household, limits parent-child interaction. While there is a policy framework for early child development [20] little has been done in terms of implementation especially for the younger pre-school children. Innovative programs are needed to educate parents and families (focusing on those with many children) on how to stimulate infants in order to optimise their socio-emotional development. In the health sector, the high utilisation of antenatal care and immunisation coupled with the availability of CHWs/VHTs provide an opportunity for integration of responsive care giving programs.

## Methodological considerations

There are a few methodological concerns in the current study. One of them is the young age group (9 to 12 months) assessed yet some disabilities may manifest later and minor disabilities may eventually change with time, especially, if addressed [28]. In addition we did not collect data to enable categorization of participants by wealth quintiles. We used the father’s occupation as proxy, which could have limited the association found compared with other studies [9, 11, 38]. In addition, the domain of Social function of the MDAT tool has some items linked to personal care, which may partially explain the findings in that domain. None the less, our results have highlighted the high prevalence of NDD, the categories of disability and the need to address it as early as possible during infancy. We further argue for the need to conduct prospective longitudinal studies of preterm babies to understand their outcomes better; the mortality and their morbidities including the NDD in Uganda and other countries with similar settings in order to design contextually appropriate interventions to prevent NDD or support infants who are affected by NDD.

## Conclusion

The study has revealed a high NDD prevalence especially in the social domain. The key factors associated with NDD are: failure to cry at birth, post-neonatal complications and mother’s parity of more than three children. Failure of a baby to cry at birth is a proxy for challenges in the quality of perinatal service provision and efforts should be made to address this as a priority area in order to minimize the risk of disability.

The post-neonatal complications suggest limited access as well as low quality service provision in the postnatal period. In resource limited settings, having a high number of children poses challenges in parent-child interaction. This calls for integration of innovative strategies in existing VHT/CHW programmes, and in ANC and immunization clinics to improve awareness of parents on opportunities for effective stimulation, child encouragement and regulation during daily nurturing to enable optimal neurodevelopment.

## Data Availability

The datasets used and/or analysed during the current study are available from the corresponding author on reasonable request.

## Abbreviations

ANC: Antenatal Care
CHW: Community Health Worker
GA: Gestational Age
HDSS: Health Demographic Surveillance Site
LBW: Low Birth Weight
LICs: Low Income Countries
LMIC: Low and Middle Income Countries
LNMP: Last normal Menstrual Period
MDAT: Malawi Developmental Assessment Tool
NDD: Neurodevelopmental Disability
VHT: Village Health Team
